# Data of epigenomic profiling of histone marks and CTCF binding sites in bovine rumen epithelial primary cells before and after butyrate treatment

**DOI:** 10.1016/j.dib.2019.104983

**Published:** 2019-12-12

**Authors:** Xiaolong Kang, Shuli Liu, Lingzhao Fang, Shudai Lin, Mei Liu, Ransom L. Baldwin, George E. Liu, Cong-jun Li

**Affiliations:** a Animal Genomics and Improvement Laboratory, BARC, Agricultural Research Service, USDA, Beltsville, MD 20705, USA; b College of Agriculture, Ningxia University, Yinchuan 750021, China; c College of Animal Science and Technology, China Agricultural University, Beijing 100193, China; d University of Edinburgh, Edinburgh EH4 2XU, United Kingdom; e College of Animal Science of South China Agricultural University, Guangzhou 510642, China; f College of Animal Science and Technology, Northwest A&F University, Shaanxi Key Laboratory of Agricultural Molecular Biology, Yangling 712100, China

**Keywords:** Butyrate, Histone marks, CTCF, Bovine rumen

## Abstract

Discovering the regulatory elements of genomes in livestock is essential for our understanding of livestock's basic biology and genomic improvement programs. Previous studies showed butyrate mediates epigenetic modifications of bovine cells. To explore the bovine functional genomic elements and the vital roles of butyrate on the epigenetic modifications of bovine genomic activities, we generated and deposited the genome-wide datasets of transcript factor binding sites of CTCF (CCCTC-binding factor, insulator binding protein), histone methylation (H3H27me3, H3K4me1, H3K4me3) and histone acetylation (H3K27ac) from bovine rumen epithelial primary cells (REPC) before and after butyrate treatment (doi: 10.1186/s12915-019-0687-8 [1]). In this dataset, we provide detailed information on experiment design, data generation, data quality assessment and guideline for data re-use. Our data will be a valuable resource for systematic annotation of regulatory elements in cattle and the functionally biological role of butyrate in the epigenetic modifications in bovine, as well as for the nutritional regulation and metabolism study of farm animal and human.

**Table:** Specifications Table

Subject area	Biochemistry, Genetics and Molecular Biology
More specific subject area	Genetics
Type of data	Table and figures
How data was acquired	ChIP-seq assay (NextSeq 500) and bioinformatics
Data format	Raw, filtered and analyzed
Experimental factors	Bovine rumen epithelial primary cells before and after butyrate treatment
Experimental features	Rumen epithelial tissue was collected from a two-week-old Holstein bull calf fed with milk replacer only. The epithelial layer of the rumen tissue was manually separated from the muscular layer and rinsed in water to remove residual feed particles. Rumen epithelial fragments generally underwent 5–6 cycles of digestion with fresh trypsin solution. 5mM of butyrate was added to the culture for 24 h before harvested. Chromatin immunoprecipitation was performed for the transcript factor binding sites of CTCF (CCCTC-binding factor, insulator binding protein), histone methylation (H3H27me3, H3K4me1, H3K4me3) and histone acetylation (H3K27ac); immunoprecipitated DNA was isolated and sequenced on Illumina NextSeq 500 platform.
Data source location	Animal Genomics and Improvement Laboratory, BARC, Agricultural Research Service, USDA, Beltsville, Maryland, USA
Data accessibility	Raw read data were deposited to NCBI Gene Expression Omnibus: GSE129423 (https://www.ncbi.nlm.nih.gov/geo/query/acc.cgi?acc=GSE129423), and data are in the related article [1].
Related research article	Fang, L., Liu, S., Liu, M., Kang, X., Lin, S. et al. Functional annotation of the cattle genome through systematic discovery and characterization of chromatin states and butyrate-induced variations. BMC biology, 2019,17 (1), 1–16. DOI: https://doi.org/10.1186/s12915-019-0687-8

**Table:** 

**Value of the Data** • A number of studies have revealed the significant roles of butyrate in diverse molecular functions and biological processes in bovine cells [2,3]. The dataset with detailed table and figure information can be used to characterize vital roles of butyrate on the epigenetic modifications of bovine cells. • The dataset showed a complex interplay between the genome and the specialized functional proteins such as CTCF, a multifunctional protein [4], as well as post-translationally modified histone markers, H3K4me1, H3K4me3, H3K27ac, and H3K27me3 [5,6]. • The dataset in our article are useful to researchers interested in butyrate function, nutritional regulation and metabolism study of farm animal and human. • This data will provide a valuable resource for systematic annotation of regulatory elements in cattle and the functionally biological role of butyrate in the epigenetic modifications in bovine.

## 1 Data

The rumen is an important organ mediating food fermentation, digest and nutrition intake in ruminants. Nutrients from dietary supplementary have been shown to influence the function of enzymes that participate in the methylation process [7,8]. Butyrate, one of the short-chain fatty acids (SCFA), can activate epigenetically-silenced genes by increasing global histone acetylation [9], as well as induces cell-cycle arrest and apoptosis [10].

**Fig. 1:**
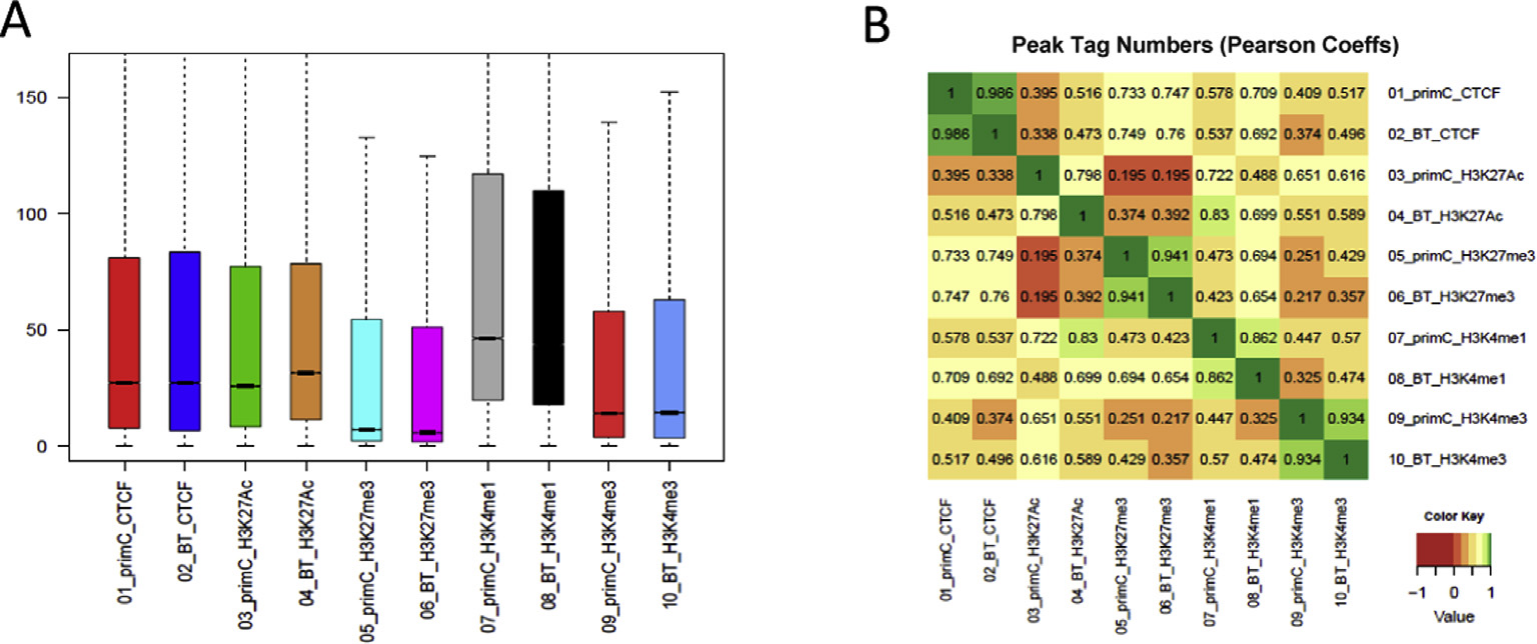
**Quality assessment of reads and ChIP signal**. (**A**) Distribution of peak tag numbers. (**B**) The Pearson correlation coefficients of all pairwise comparisons. Rumen-primC (PC): rumen-primary epithelial cells; Rumen-BT (BT): rumen primary epithelial cells treated with butyrate.

**Fig. 2:**
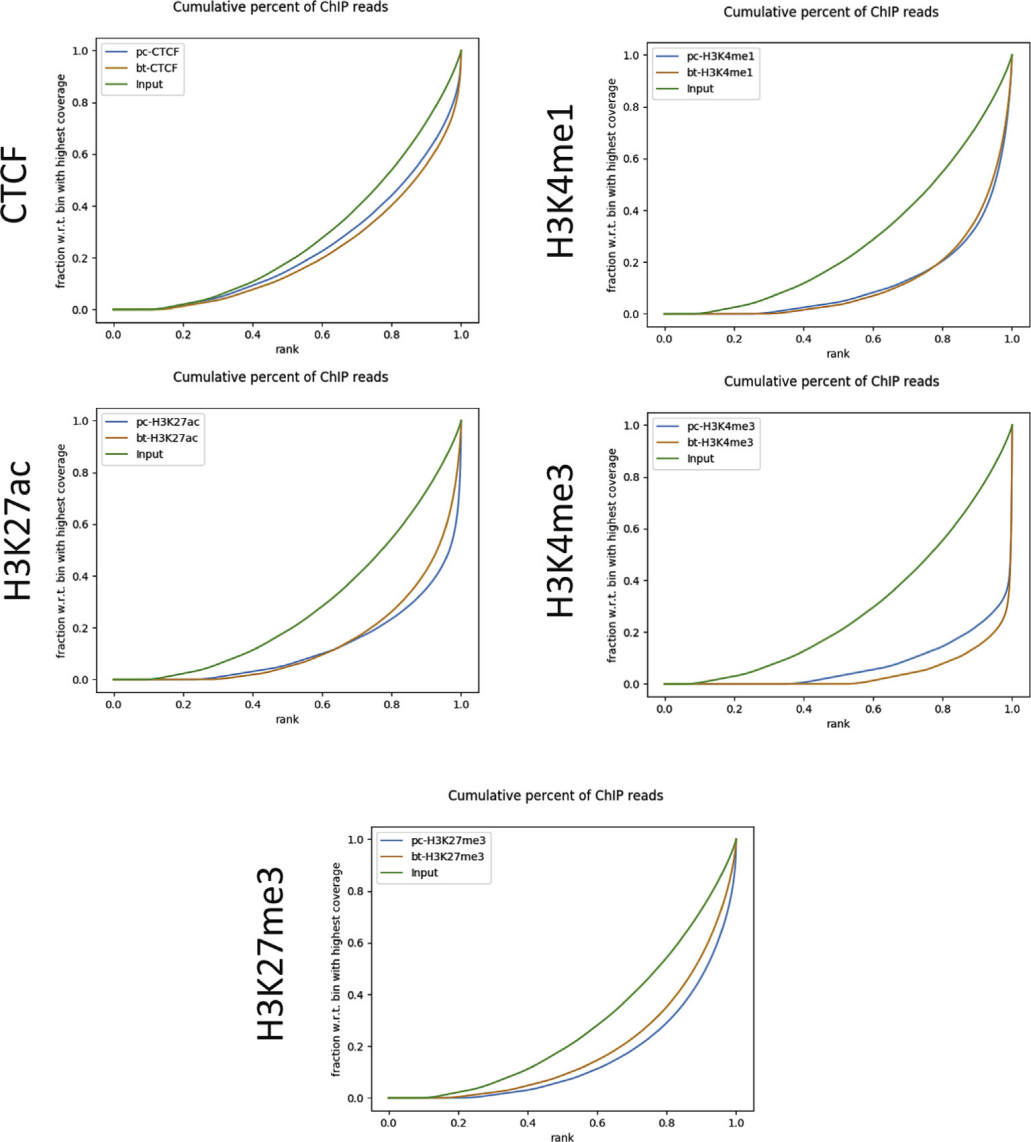
**Cumulative read coverage.** A specific and strong ChIP enrichment was indicated by a steep rise of the cumulative sum towards the highest rank. x-axis: percentage rank of signal enriched. y-axis: fraction of cumulative tag density.

The data of this article sought to investigate the global profile of binding sites of CTCF and four histone marks (H3K4me1, H3K4me3, H3K27ac, and H3K27me3) in bovine rumen epithelial primary cells before and after butyrate treatment by chromatin immunoprecipitation followed by next-generation sequencing (ChIP-seq). CTCF is a DNA binding factor with defined functions of regulation of gene expression (transcription activation and repression); RNA splicing, and enhancer/promotor insulation [4]. A total of 468,849,656 raw reads were generated by Illumina sequencing (NextSeq 500), with an average of 39,310,948 ± 2,881,720 per sample (Table 1). The raw reads files (fastq format) of each sample have been uploaded to the NCBI Gene Expression Omnibus (NCBI Gene Expression Omnibus GSE129423, total 11 samples in the dataset, samples ID were GSM3712486-GSM3712696). The sequencing statistics raw reads and alignment for each dataset were summarized in Table 1. After trimming of raw reads, an average of 23 million reads was mapped uniquely to the bovine genome, and 23,401,740 ± 4,603,827 final tags were generated for further analysis. After normalization for tags for each library, 19,627,913 tags used for peak calling for each sample (Table 1). Detailed information on sequence quality control was summarized (Fig. 1 and Supplementary Fig. 1). Boxed area represents the central 2 quartiles (middle line means median), while the whiskers show the top and bottom quartiles without outliers (Fig. 1A). Then heatmap was employed to show the Pearson correlation coefficients (r) of all pairwise comparisons (Fig. 1B). Three different charts were generated to compare the peak sizes and strength between butyrate-treated and untreated samples (Fig. 1, Fig. 2 and Supplementary Fig. 2). For each pairwise comparison, a scatter plot was generated by plotting the tag numbers of sample 1 against sample 2 for each merged region (Supplementary Fig. 2 and Supplementary Table S1). The slope was a measure for the average ratio in tag numbers between butyrate-treated and untreated samples. Peak size boxplot was used for comparing the distribution of peak tag numbers between the samples. The metric used for these charts was the number of tags in the merged peak regions of the assay. The number of tags was calculated from the average values by taking into account the length of the merged regions, the bin size and the in-silico extension (Supplementary Table S1).

**Table 1:** Sequencing read alignment statistics for ChIP-seq data set.

	Total number of reads	Total number of alignments	Unique alignments (without duplicate reads)	Unique alignments %	Final number of tags	Normalized tags	Input tags used for peak calling	FRIP (%)
PC_CTCF	39,610,540	34,652,442	23,328,713	67.3	23,205,192	23,205,192	19,627,913	13.9
BT_CTCF	40,990,109	37,448,576	23,331,071	62.3	23,225,349	23,205,192	19,627,913	19.4
PC_H3K27ac	42,565,192	36,737,369	24,488,622	66.7	24,412,621	20,565,887	19,627,913	39.3
BT_H3K27ac	48,050,969	44,040,540	20,627,722	46.8	20,565,887	20,565,887	19,627,913	23.2
PC_H3K27me3	43,699,377	40,031,049	26,146,058	65.3	26,053,236	26,053,236	19,627,913	49.0
BT_H3K27me4	42,961,510	40,131,792	28,969,252	72.2	28,861,259	26,053,236	19,627,913	45.6
PC_H3K4me1	43,243,959	40,767,839	33,001,733	81.0	32,915,813	32,783,103	19,627,913	27.2
BT_H3K4me2	46,973,975	43,593,617	32,865,978	75.4	32,783,103	32,783,103	19,627,913	20.0
PC_H3K4me3	41,860,309	38,598,738	23,586,847	61.1	23,473,852	21,092,832	19,627,913	60.7
BT_H3K4me4	38,952,658	35,316,748	21,164,467	59.9	21,092,832	21,092,832	19,627,913	68.8
Input	39,941,058	37,593,762	19,832,143	52.8	19,627,913	19,627,913

**Fig. 3:**
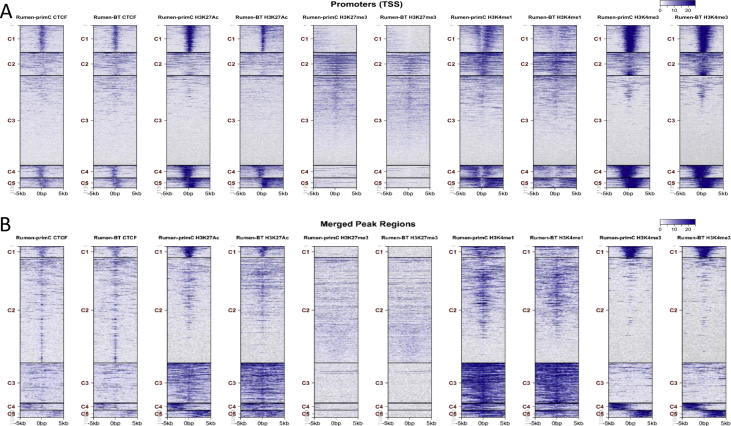
**Genome-wide enrichment of peaks for histone marks and CTCF**. (**A**) Heatmap of tag distributions across promoters (TSS, Transcription Start Sites) (default = 5 clusters; indicated by C1–C5, values in z-axis/color, regions in y-axis). (**B**) Heatmap of tag distributions across merged regions. The gradient blue-to-white color indicates high-to-low count in the corresponding region. Rumen-primC (PC): rumen-primary epithelial cells; Rumen-BT (BT): rumen primary epithelial cells treated with butyrate.

**Fig. 4:**
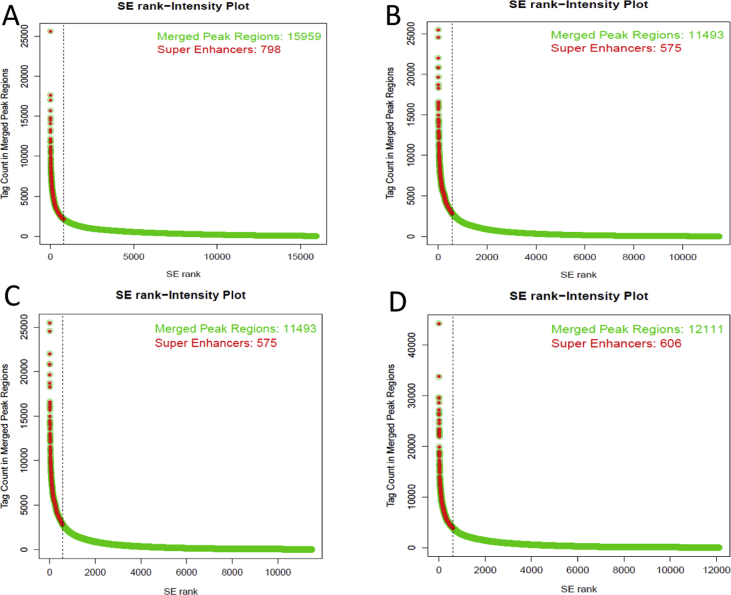
**Identification of Super-Enhancers.** Enhancers are plotted in decreasing order based on ChIP-Seq peak intensity (Tag count). X-axis: Number of Merged peak regions. Y-axis: Tag counts in merged peak regions. Super-Enhancers for both H3K27ac and H3K4me1 before and after butyrate treatment were showed in a-d, separately. primC-: rumen-primary epithelial cells; BT-: rumen primary epithelial cells treated with butyrate.

The cumulative read coverage for each sample plotted by the fingerprint program from deeptools (v3.3.0) [11] was provided (Fig. 2). Peak distributions across the genomic regions were displayed with pie plots (Supplementary Fig. 3). Tag distributions (using bigWig metrics) across all merged regions (= all peak regions), transcription start sites (TSS) or gene bodies were determined and presented either as average plots (average of values for all target regions) (Supplementary Fig. 4) or as heatmaps (values in z-axis/color, regions in y-axis) (Fig. 3). Overlapping intervals are grouped into “Merged Regions” to compare peak metrics between 2 or more samples (Supplementary Table S2). Super-enhancers were identified by using a proprietary algorithm as described previously [12]. First, MACS [13] or SICER [13] peaks generated by the standard ChIP-Seq analyses were merged if their inner distance was equal or less than 12,500 bp. Then, the merged peak regions with the strongest signals (top 5%) were identified as Super-enhancers (Fig. 4).

## 2 Experimental design, materials, and methods

### 2.1 Animal and tissue collection

Animal care and tissue isolation work were approved by the Beltsville Area Animal Care and Use Committee Protocol Number 07-025. The methods for epithelial cell isolation and culture were described in an earlier report [14]. Rumen epithelial tissue was collected from a two-week-old Holstein bull calf fed with milk replacer only. At sacrifice, rumen epithelial tissue was photographed and collected from the anterior portion of the ventral sac of the rumen beneath the reticulum and below the rumen fluid layer. The epithelial layer of the rumen tissue was manually separated from the muscular layer and rinsed in water to remove residual feed particles. Samples were further rinsed in ice-cold saline. The tissue was added to 50 ml digestion solution (2% trypsin and 1.15 mmol CaCl_2_ in phosphate-buffered saline) and then was incubated in 37 °C incubator for 15 min.

Rumen epithelial fragments generally underwent 5–6 cycles of digestion with fresh trypsin solution. The first two rounds of digestion were discarded, and the third, fourth and fifth rounds of digestion were collected. After the epithelial tissue had undergone trypsin digestion, the solution was filtered through a 300-μm-nylon mesh. Following filtration, cell fractions were centrifuged at 60×*g* for 5 min at 4 °C to pellet the rumen cells. Cells then subjected to three wash cycles with sterile PBS with antibiotic-antimycotic (100 units/ml of Penicillin G sodium, streptomycin sulfate, 0.25μg amphotericin B as Fungizone). Cells were counted using a hemacytometer, and cell viabilities were estimated by trypan blue dye exclusion assays. Cells were plated in a 25 cm plate at a density of 1 million cells/dish in DMEM with antibiotic-antimycotic and 5% fetal bovine serum (DMEM-FBS). After 24h in culture, the cell media were removed and replaced with fresh DMEM-FBS. Cell media were changed every 48h until the cells reached confluence (4–7 days). Cells then removed from the dish by trypsinization, quantified and reseeded for treatment or frozen in liquid nitrogen for further culture. To test the response of the primary rumen epithelial cells to the treatment of butyrate, 5mM of butyrate was added to the culture for 24 h before harvested.

### 2.2 ChIP sequencing preparation

ChIP-seq of rumen epithelial tissue was performed as reported in our earlier publication [15]. In short, DNA recovered from a conventional ChIP procedure was quantified using the QuantiFluor fluorometer (Promega, Madison, WI). DNA integrity was verified using the Agilent Bioanalyzer 2100 (Agilent; Palo Alto, CA, USA). The DNA was then processed, including end repair, adaptor ligation, and size selection, using an Illumina sample prep kit following the manufacturer**'**s instructions (Illumina, San Diego, CA, USA). Final DNA libraries were validated and sequenced at 75-nt per sequence read, using an Illumina NextSeq 500 platform.

### 2.3 Read mapping and quality control

The quality of base calling for raw reads generated by Illumina sequencer was assessed using the FastQC program (https://www.bioinformatics.babraham.ac.uk/projects/fastqc/,v0.11.4) to ensure that there are no biases or problem in our raw data. The trimmed reads were aligned to the bovine reference genome (BosTau_UMD3.1) using the BWA algorithm with default settings [16]. After de-duplication, only reads that pass Illumina's purity filter, align with no more than 2 mismatches, and map uniquely to the genome were used in the subsequent analysis. To identify the density of fragments (extended tags) along the genome, the genome was divided into 32-nt bins and the number of fragments in each bin is determined. To compare peak metrics between 2 or more samples, overlapping intervals are grouped into “Merged Regions” by Samtools (v1.9) [13]. Deeptools (v3.3.0) [11] was used to plot the cumulative read coverage for each sample. We used the default versions of code to process our datasets. All sequenced data were aligned by the BWA algorithm and peaks were detected by MACS(v2.1.0) [13] (CTCF, H3K27ac, H3K4me1, H3K4me3) and SICER(v1.1) [13] (H3K27me3). Graphics were generated using seqplot R bioconductor package and deeptools [11].
