# Supplementary figures and images for: Data of epigenomic profiling of histone marks and CTCF binding sites in bovine rumen epithelial primary cells before and after butyrate treatment
Source: Data Brief. 2019 Dec 12;28:104983. doi: 10.1016/j.dib.2019.104983 (PMC6933192; doi:10.1016/j.dib.2019.104983)

# PC

# BT

## CTCF

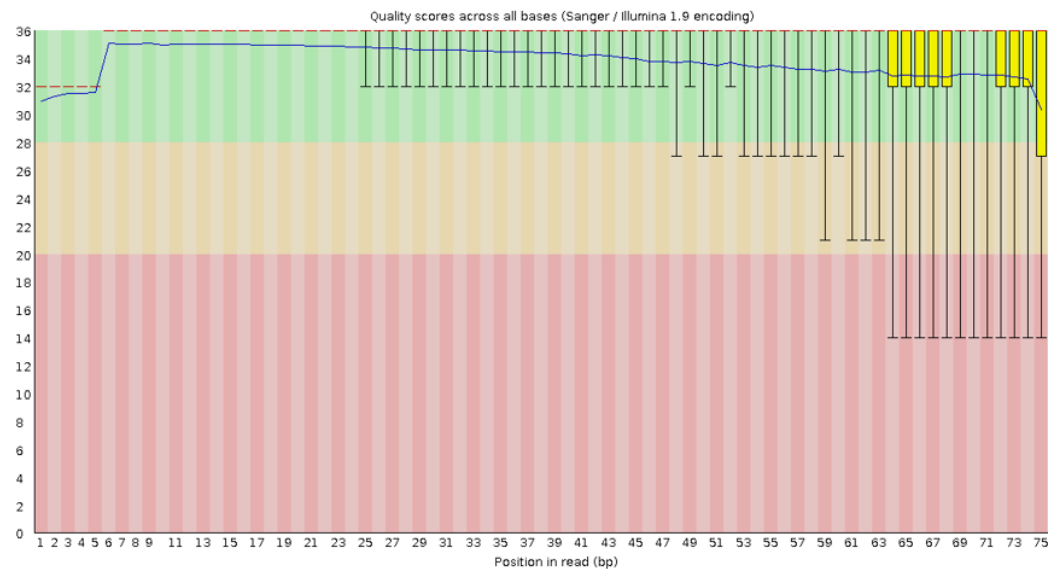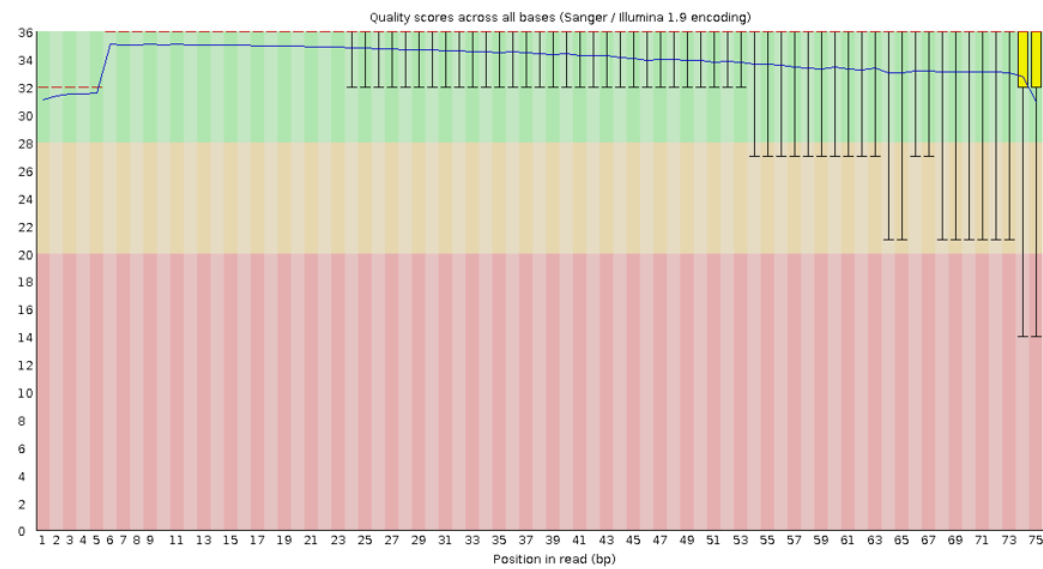

## H3K27ac

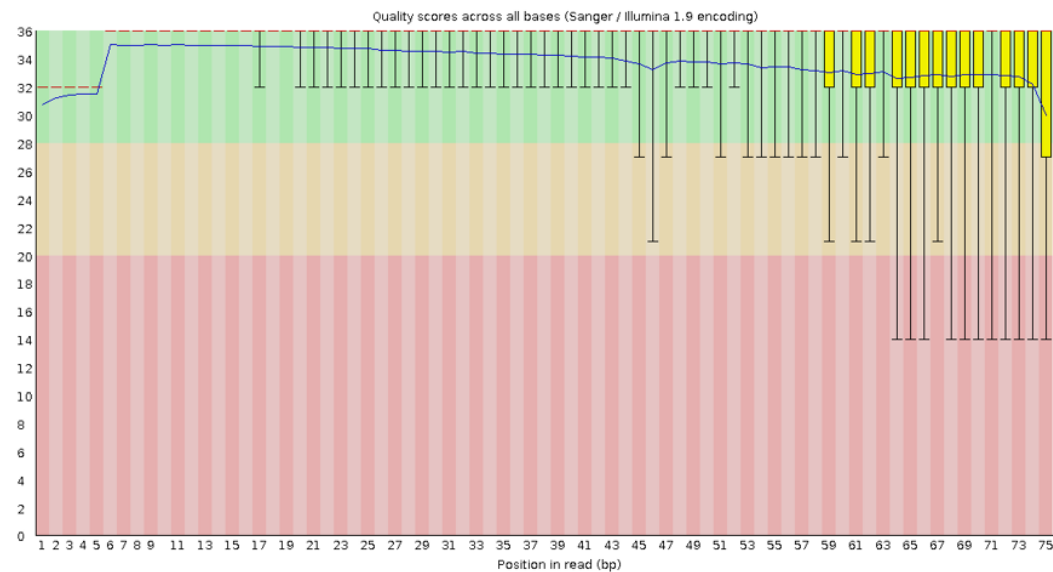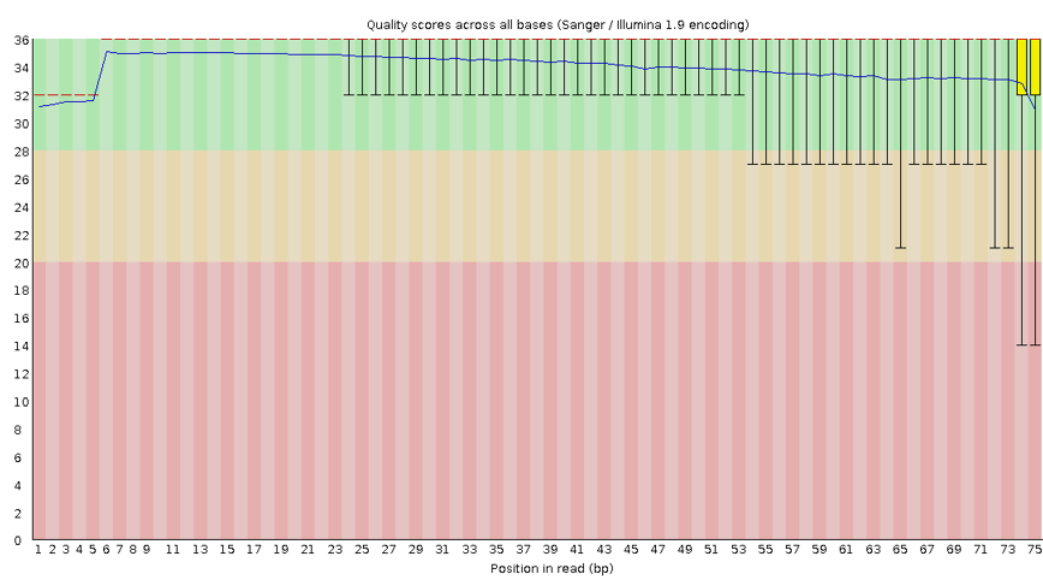

PC

H3K27me3

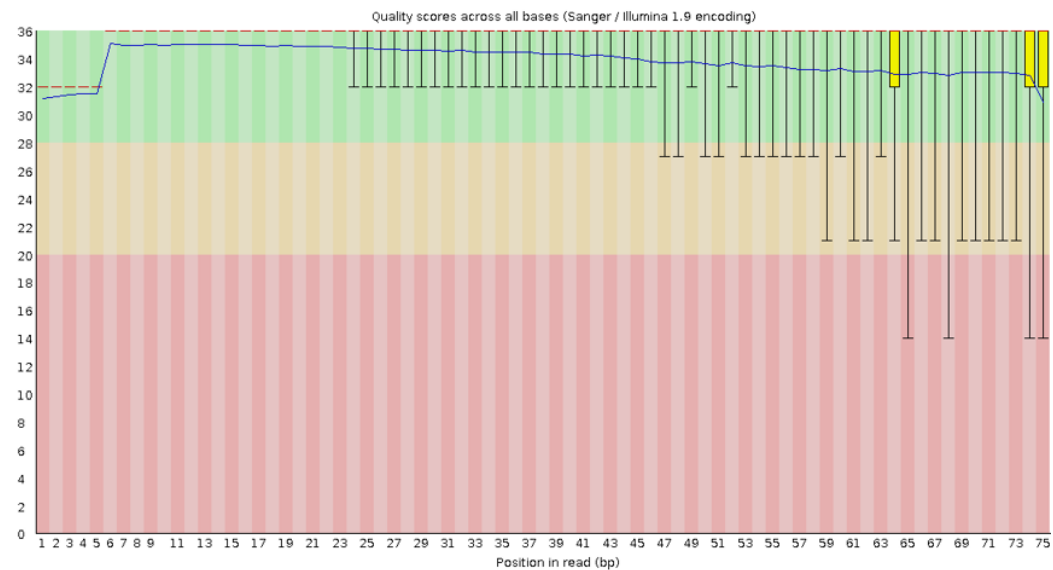

BT

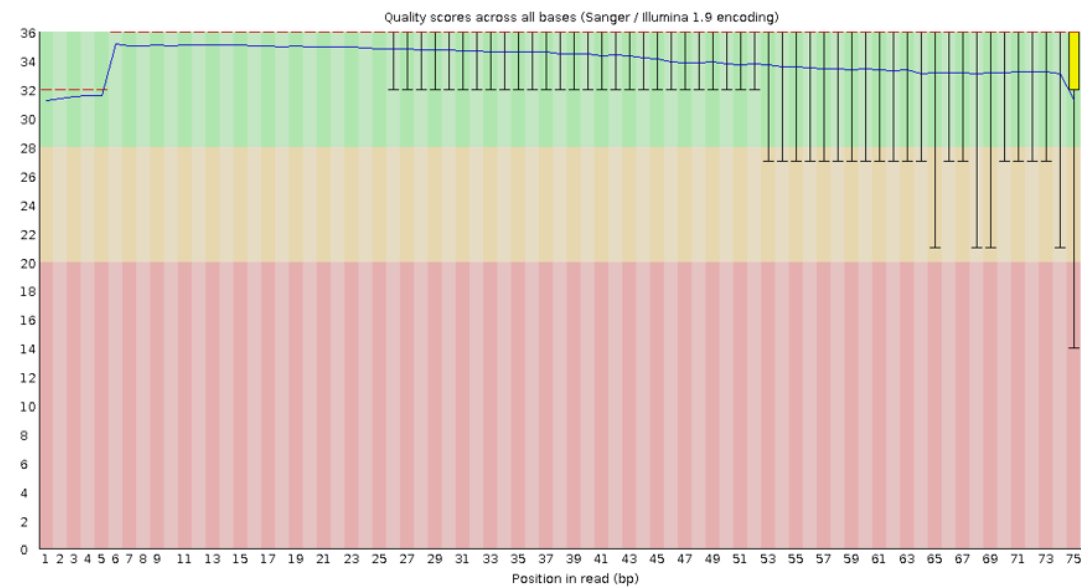

H3K4me1

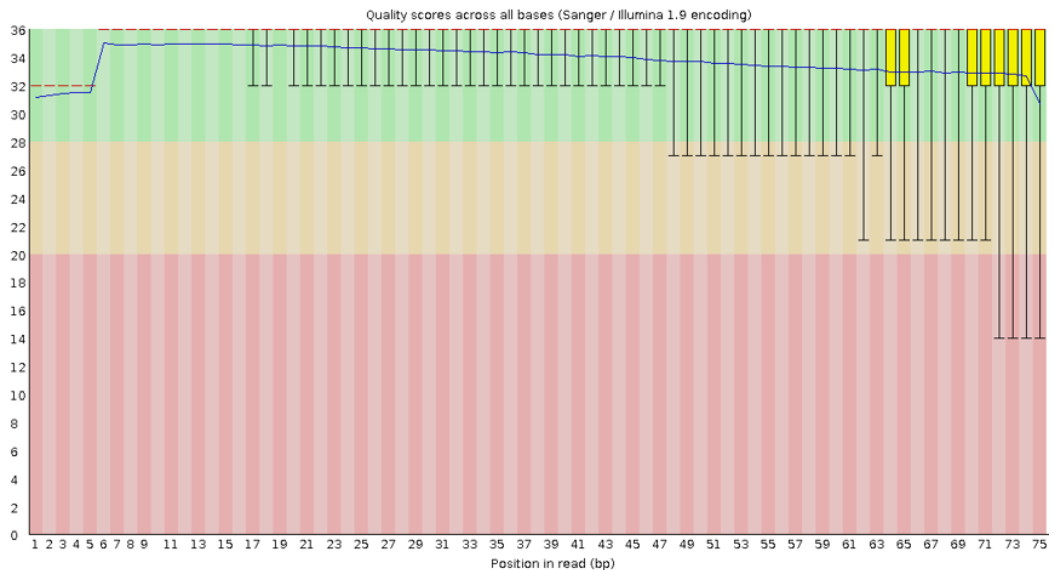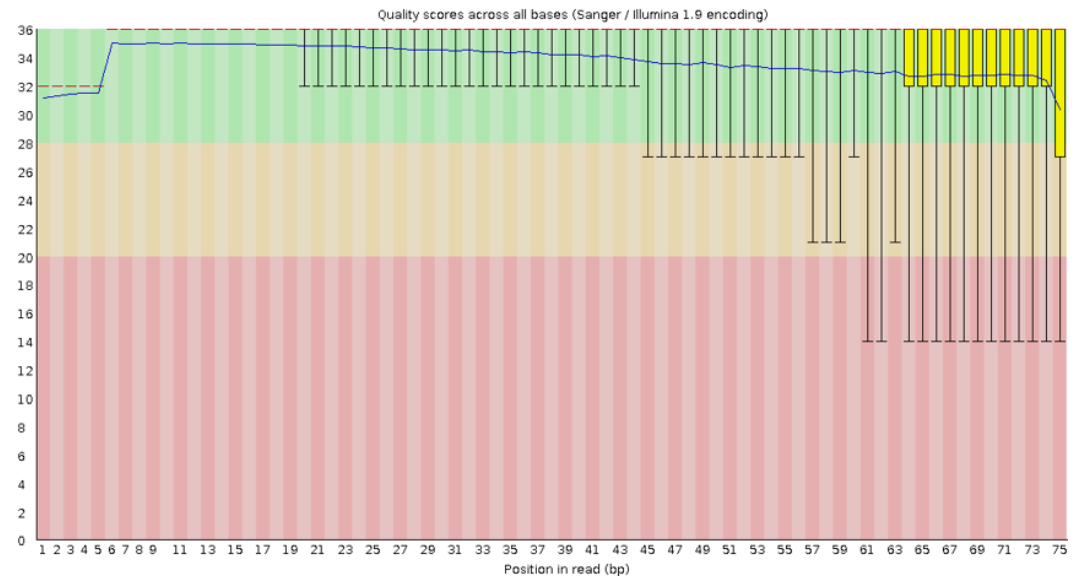

# PC

# BT

## H3K4me3

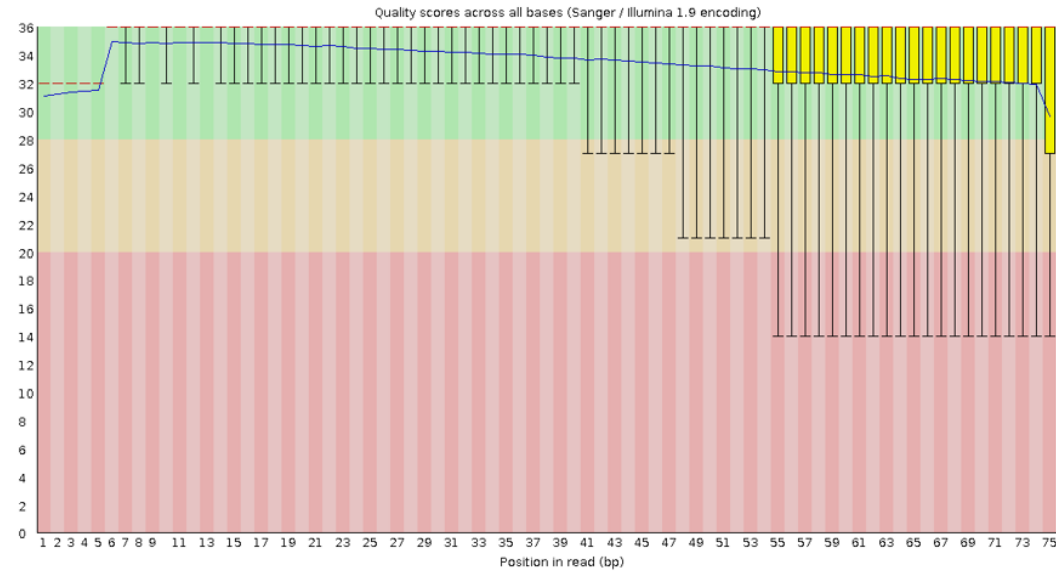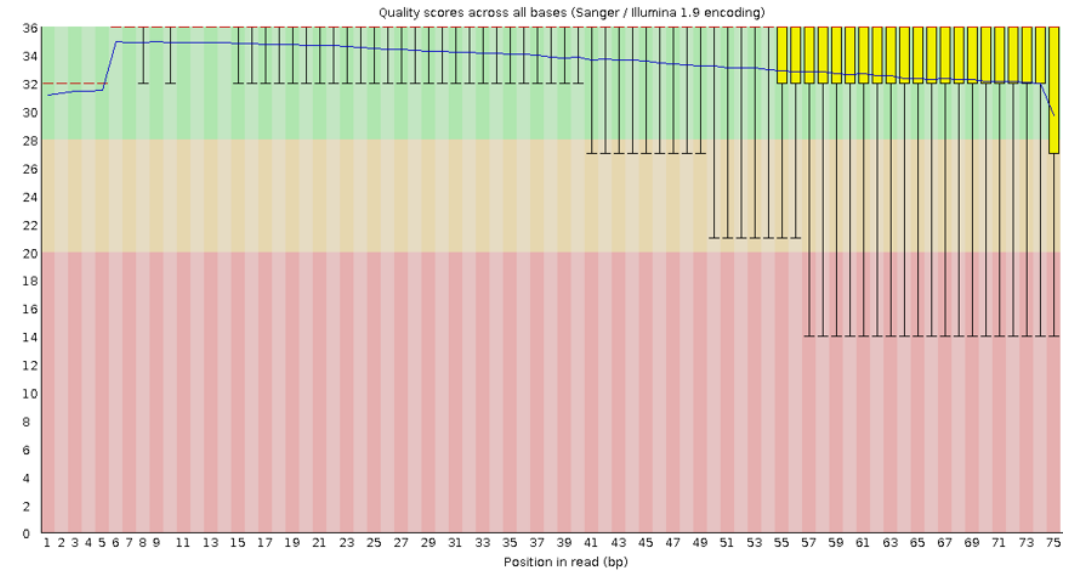

## Input

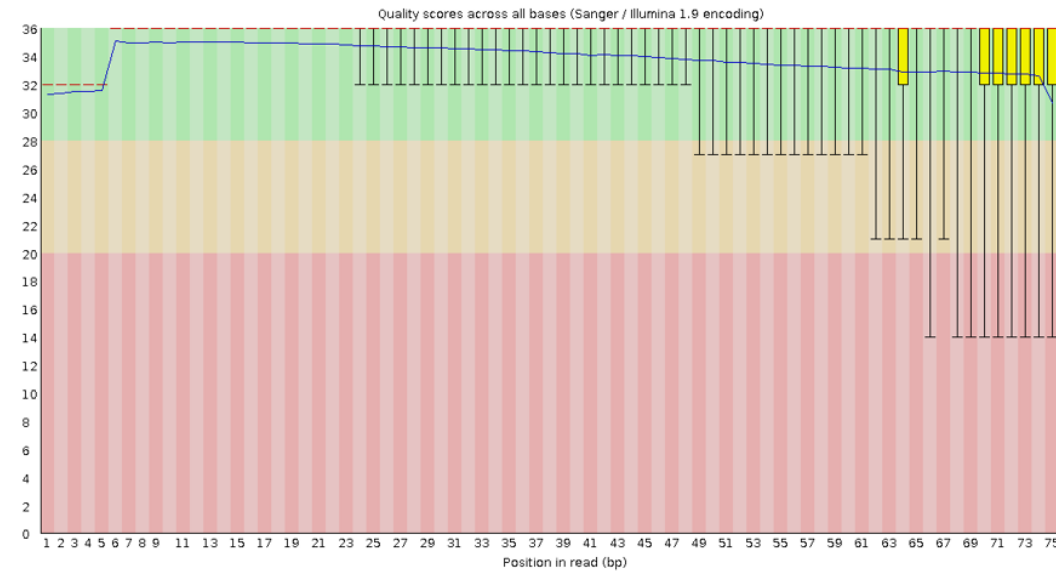

Supplement: Multimedia component 1 [file mmc1.pdf]

## H3K4me1

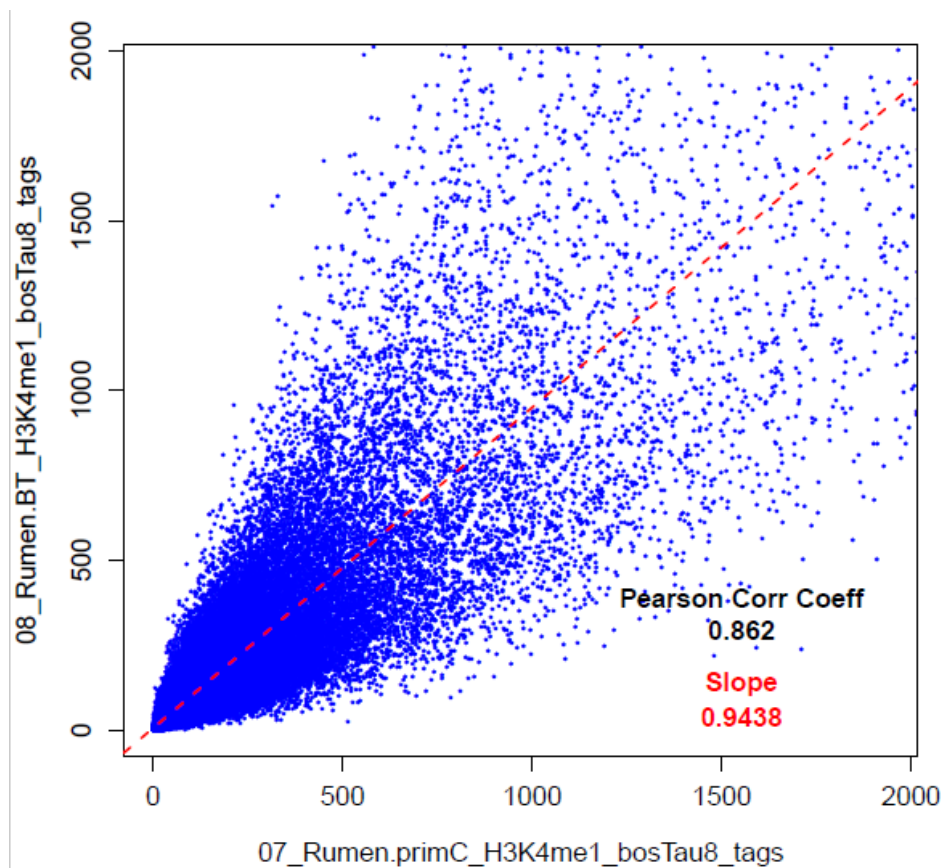

## H3K4me3

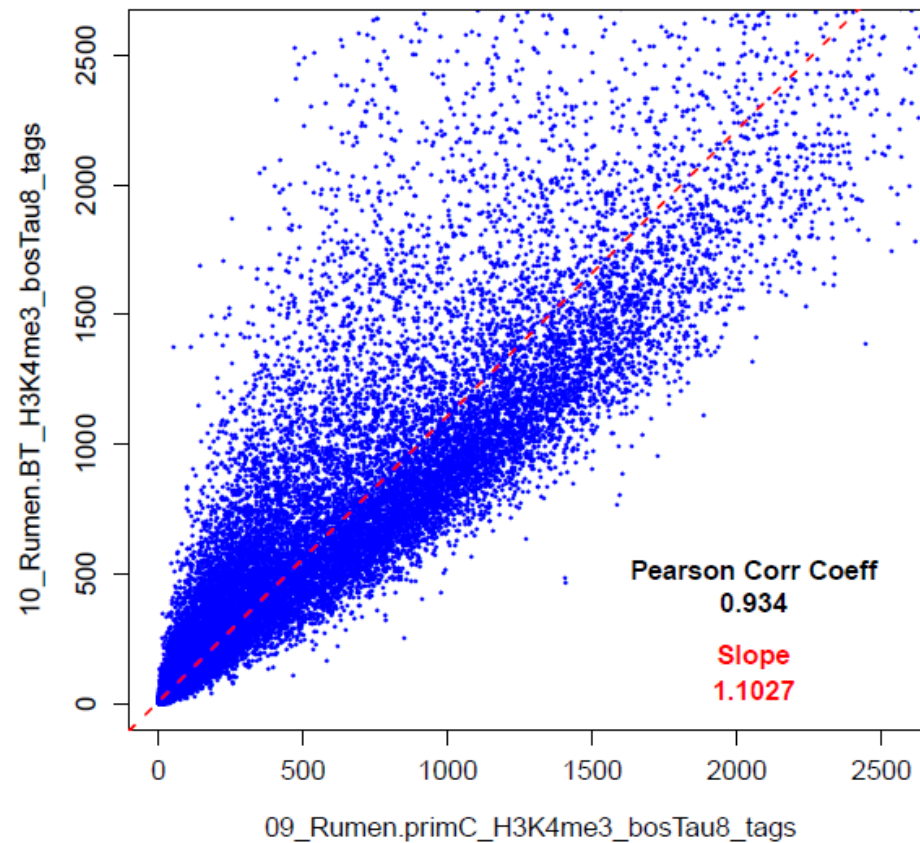

## H3K27me3

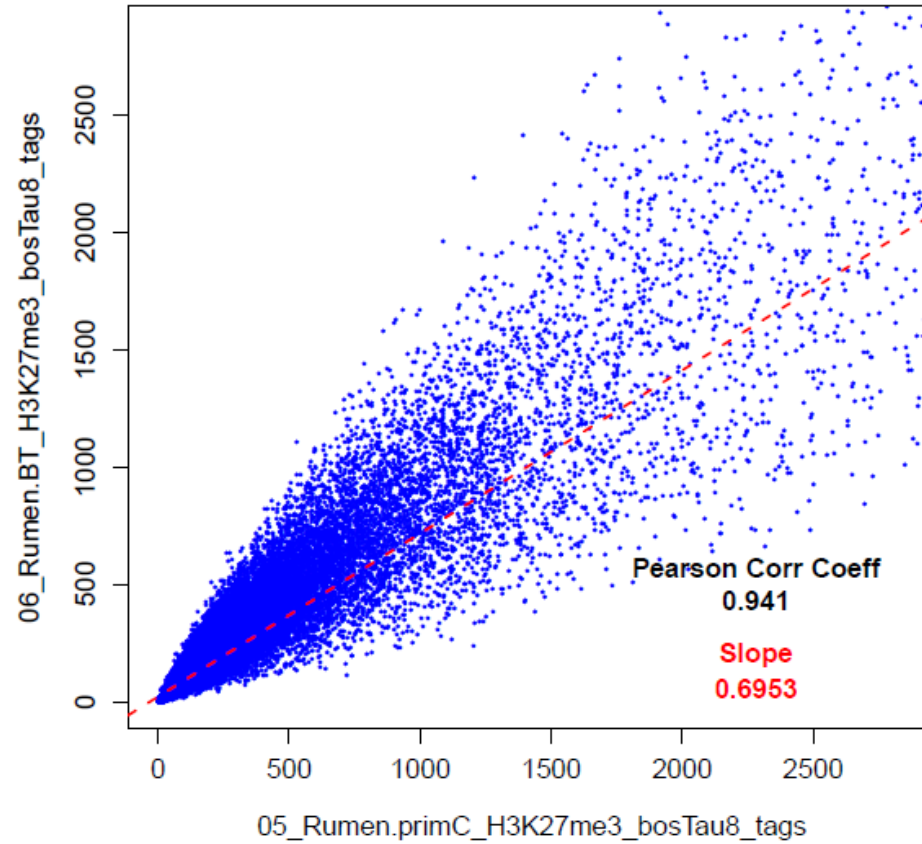

## H3K27ac

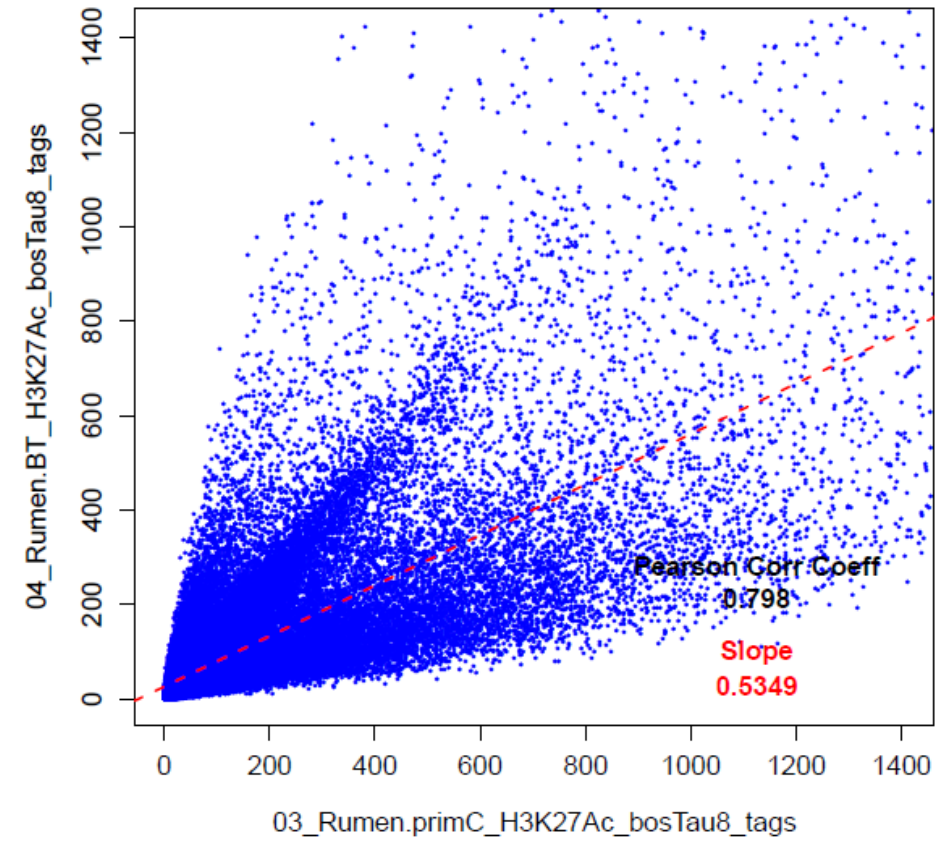

# CTCF

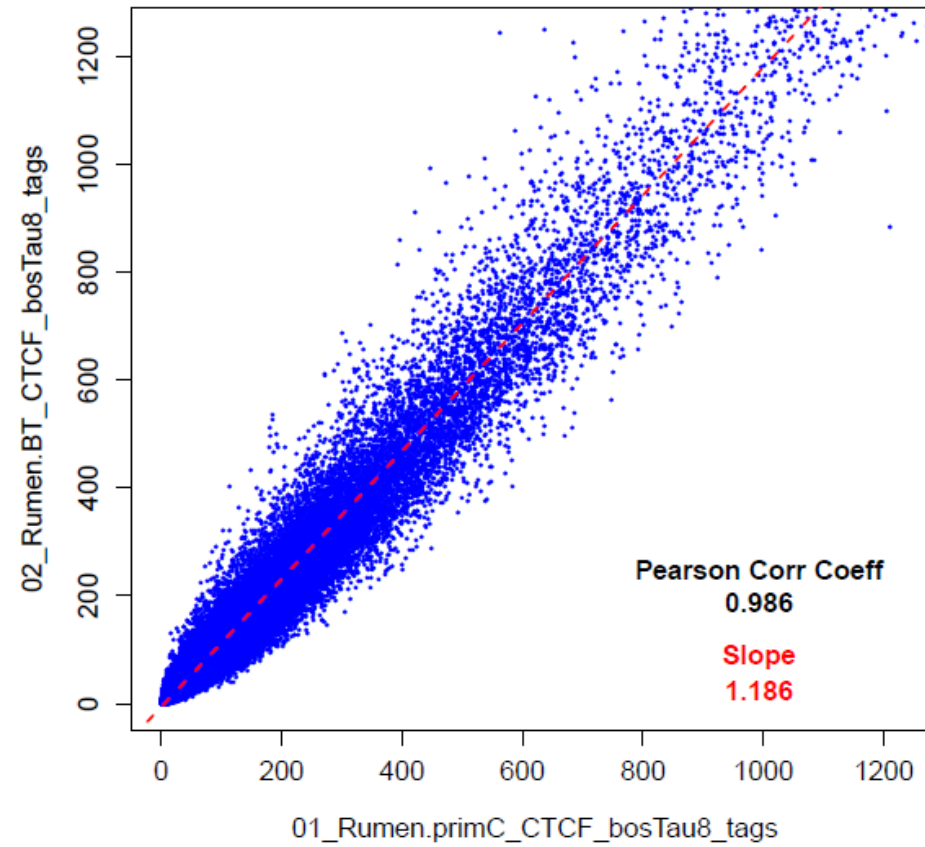

Supplement: Multimedia component 2 [file mmc2.pdf]

Promoters (TSS)

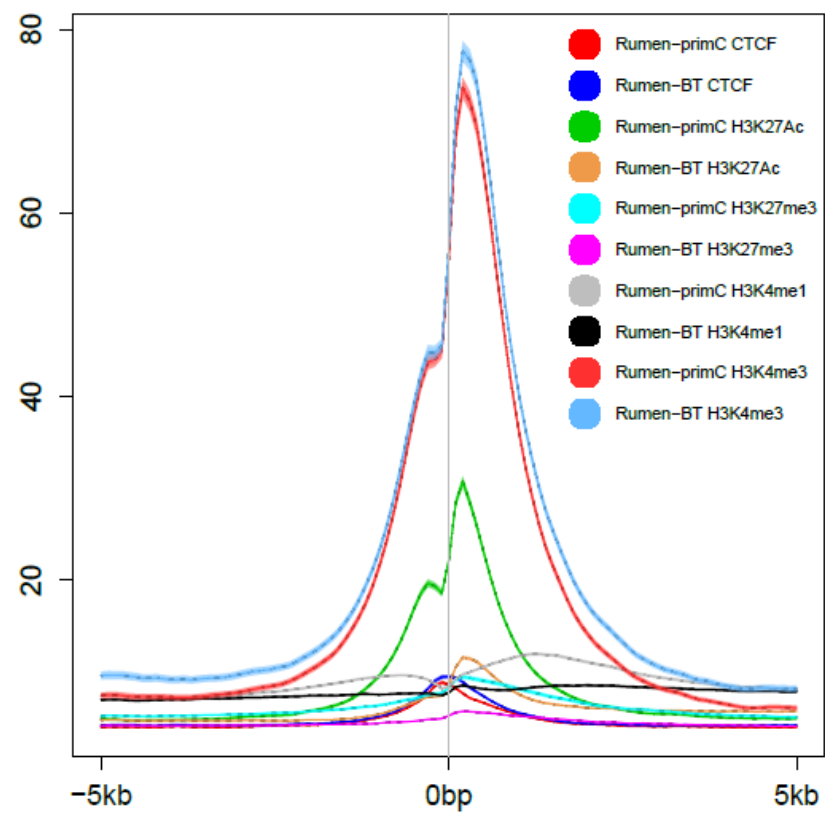

Merged Peak Regions

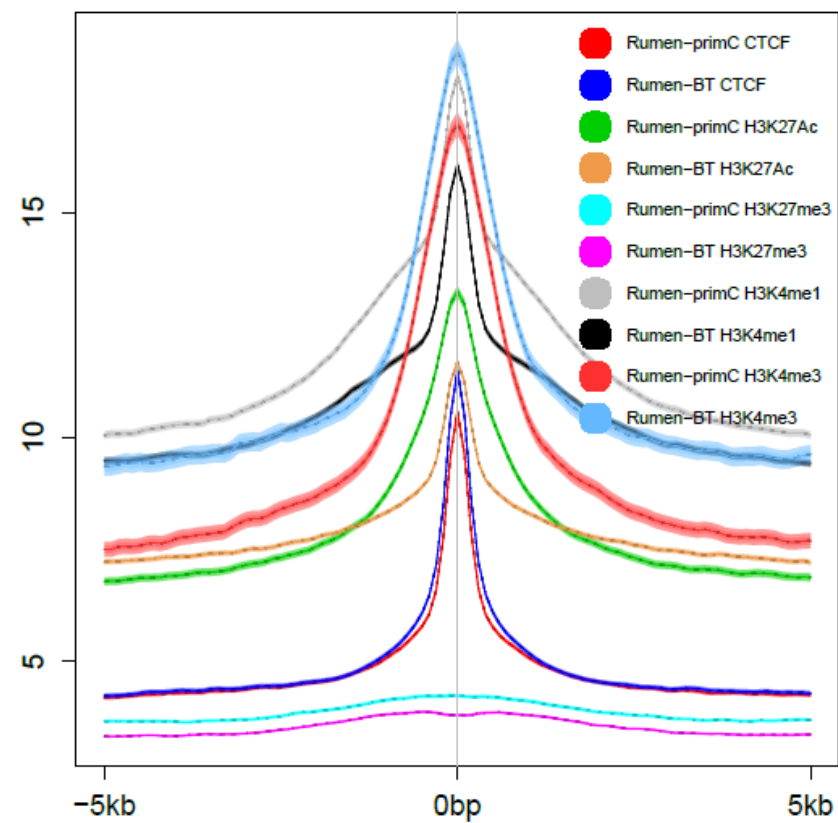

## Genebodies

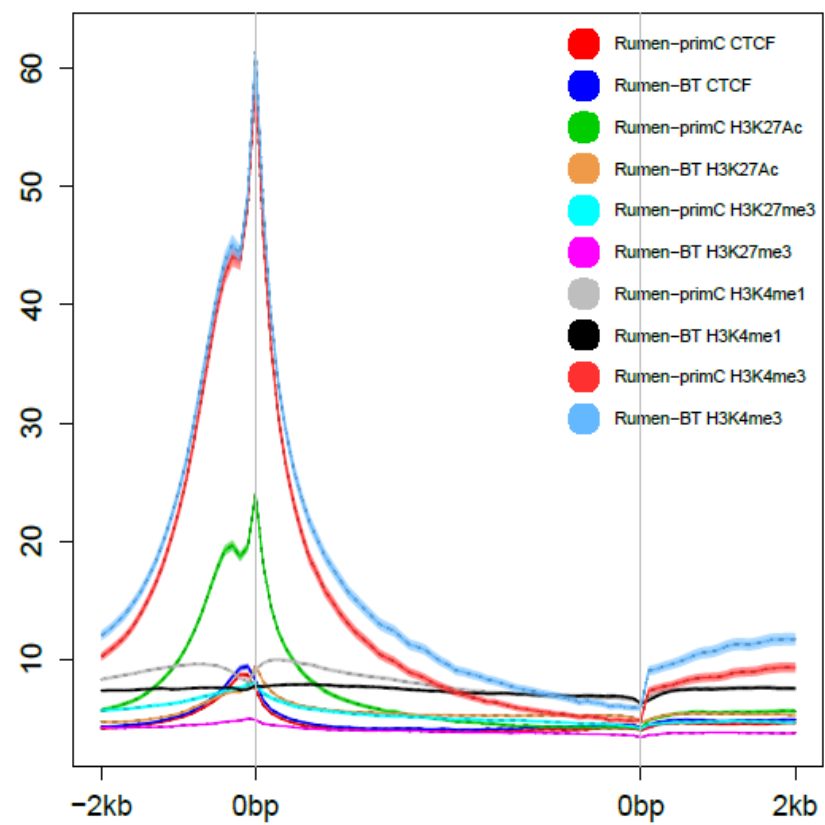

Supplement: Multimedia component 4 [file mmc4.pdf]
