# Supplementary material for: Data of epigenomic profiling of histone marks and CTCF binding sites in bovine rumen epithelial primary cells before and after butyrate treatment
Source: Data Brief. 2019 Dec 12;28:104983. doi: 10.1016/j.dib.2019.104983 (PMC6933192; doi:10.1016/j.dib.2019.104983)

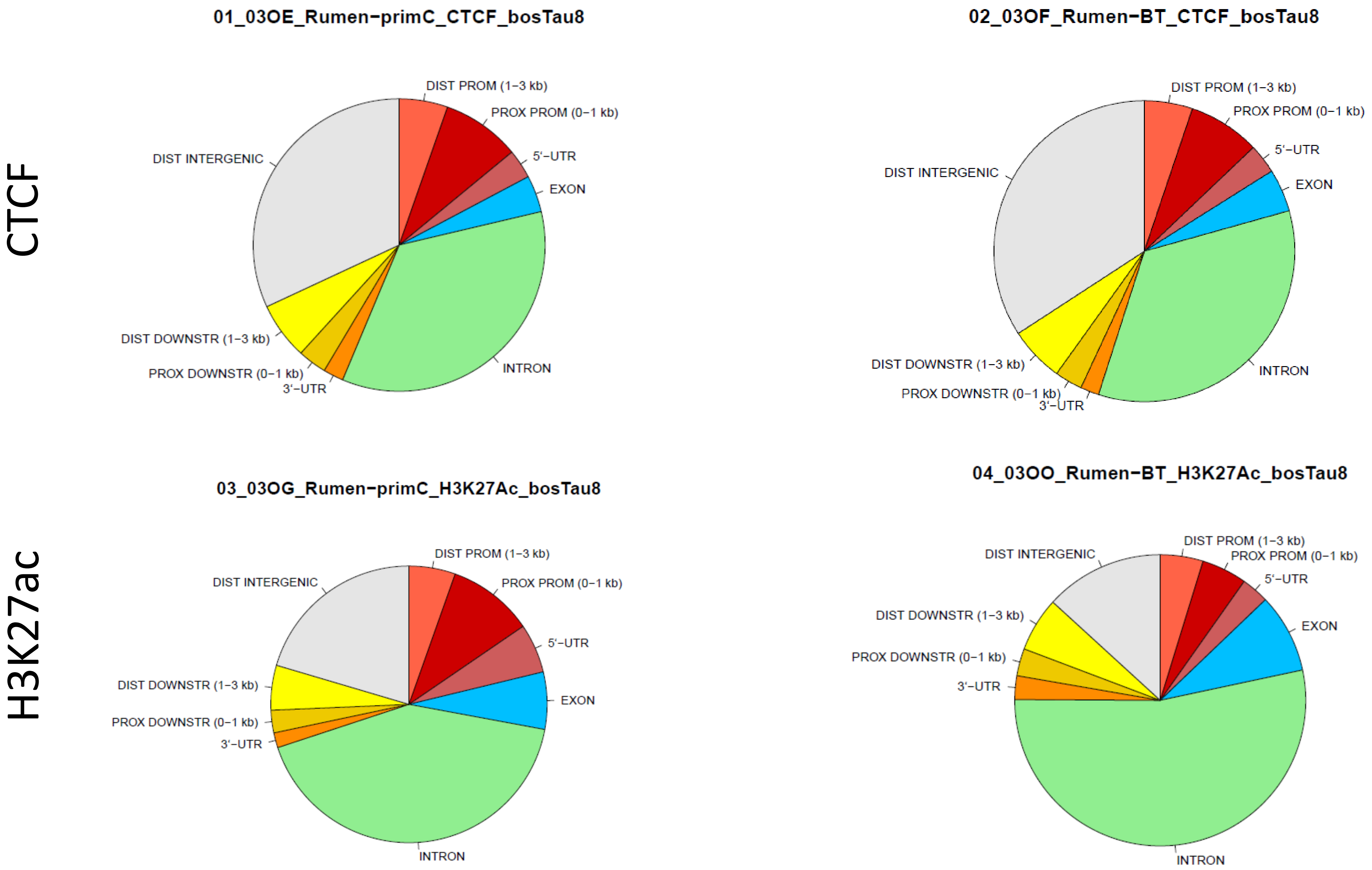
CTCF
H3K27ac
01_03OE_Rumen−primC_CTCF_bosTau8
DIST PROM (1−3 kb)
PROX PROM (0−1 kb)
5'−UTR
EXON
INTRON
3'−UTR
PROX DOWNSTR (0−1 kb)
DIST DOWNSTR (1−3 kb)
DIST INTERGENIC
02_03OF_Rumen−BT_CTCF_bosTau8
DIST PROM (1−3 kb)
PROX PROM (0−1 kb)
5'−UTR
EXON
INTRON
3'−UTR
PROX DOWNSTR (0−1 kb)
DIST DOWNSTR (1−3 kb)
DIST INTERGENIC
03_03OG_Rumen−primC_H3K27Ac_bosTau8
DIST PROM (1−3 kb)
PROX PROM (0−1 kb)
5'−UTR
EXON
INTRON
3'−UTR
PROX DOWNSTR (0−1 kb)
DIST DOWNSTR (1−3 kb)
DIST INTERGENIC
04_03OO_Rumen−BT_H3K27Ac_bosTau8
DIST PROM (1−3 kb)
PROX PROM (0−1 kb)
5'−UTR
EXON
INTRON
3'−UTR
PROX DOWNSTR (0−1 kb)
DIST DOWNSTR (1−3 kb)
DIST INTERGENIC

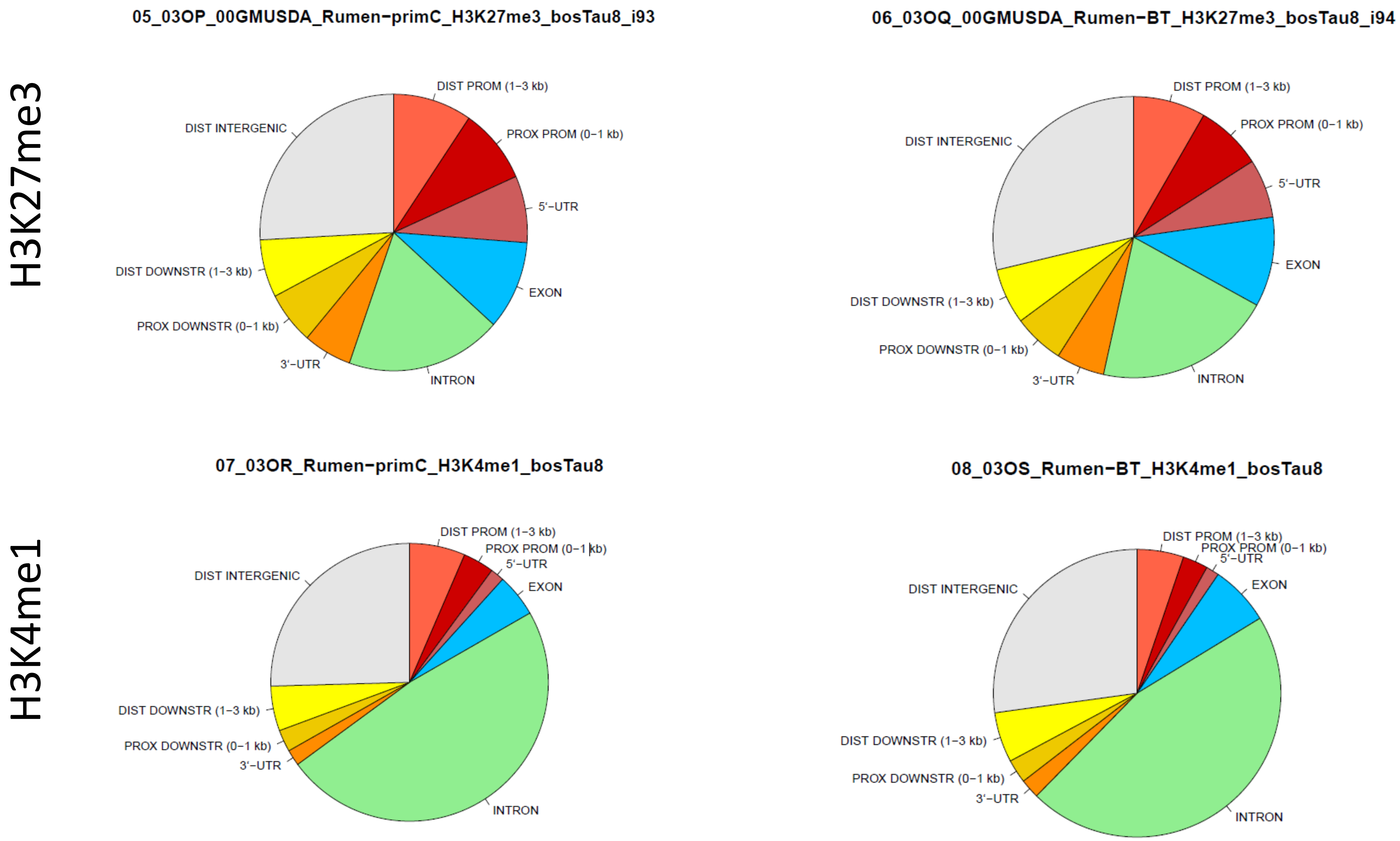
H3K27me3
05_03OP_00GMUSDA_Rumen−primC_H3K27me3_bosTau8_i93
DIST PROM (1−3 kb)
PROX PROM (0−1 kb)
5'−UTR
EXON
INTRON
3'−UTR
PROX DOWNSTR (0−1 kb)
DIST DOWNSTR (1−3 kb)
DIST INTERGENIC
06_03OQ_00GMUSDA_Rumen−BT_H3K27me3_bosTau8_i94
DIST PROM (1−3 kb)
PROX PROM (0−1 kb)
5'−UTR
EXON
INTRON
3'−UTR
PROX DOWNSTR (0−1 kb)
DIST DOWNSTR (1−3 kb)
DIST INTERGENIC
H3K4me1
07_03OR_Rumen−primC_H3K4me1_bosTau8
DIST PROM (1−3 kb)
PROX PROM (0−1 kb)
5'−UTR
EXON
INTRON
3'−UTR
PROX DOWNSTR (0−1 kb)
DIST DOWNSTR (1−3 kb)
DIST INTERGENIC
08_03OS_Rumen−BT_H3K4me1_bosTau8
DIST PROM (1−3 kb)
PROX PROM (0−1 kb)
5'−UTR
EXON
INTRON
3'−UTR
PROX DOWNSTR (0−1 kb)
DIST DOWNSTR (1−3 kb)
DIST INTERGENIC

H3K4me3

09_03P3_Rumen−primC_H3K4me3_bosTau8

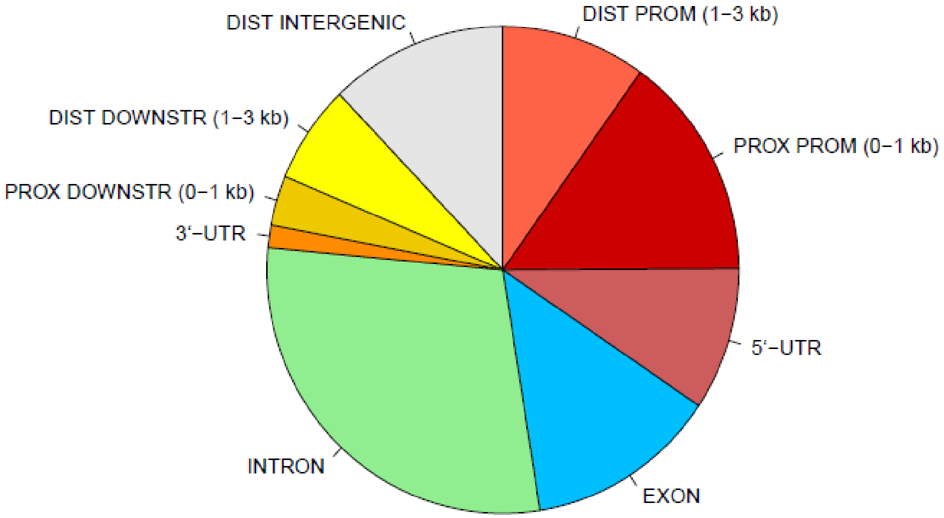

10_03P4_Rumen−BT_H3K4me3_bosTau8

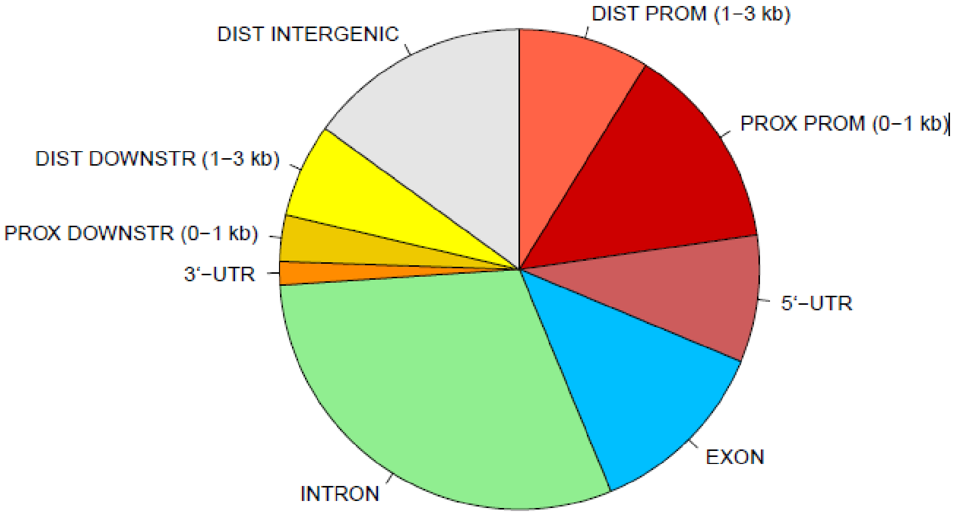

Supplement: Multimedia component 3 [file mmc3.pdf]
